# Diet crude protein reduction on follicular fluid and cumulus-oocyte complexes of mid-lactating Girolando cows

**DOI:** 10.1590/1984-3143-AR2021-0088

**Published:** 2022-09-12

**Authors:** Luciano de Rezende Carvalheira, Gustavo Bervian dos Santos, Jasmim, Clóvis Ribeiro Guimarães, Mariana Magalhães Campos, Fernanda Samarini Machado, Alexandre Mendonça Pedroso, Tadeu Eder da Silva, Luiz Altamiro Garcia Nogueira, André Luís Rios Rodrigues, Bruno Campos de Carvalho

**Affiliations:** 1 Faculdade de Veterinária, Universidade Federal Fluminense, Niterói, RJ, Brasil; 2 Núcleo Multidisciplinar de Pesquisa em Biologia, Universidade Federal do Rio de Janeiro, Duque de Caxias, RJ, Brasil; 3 Escola de Veterinária, Universidade Federal de Minas Gerais, Belo Horizonte, MG, Brasil; 4 Embrapa Gado de Leite, Juiz de Fora, MG, Brasil; 5 AMP Consultoria, Piracicaba, SP, Brasil; 6 Department of Animal and Dairy Science, University of Wisconsin-Madison, Madison, WI, USA

**Keywords:** apoptosis, nutrition, reproduction, urea

## Abstract

This study evaluated the effect of crude protein (CP) reduction in four diets (156, 139, 132, and 127 g Kg^-1^ DM) maintaining constant metabolizable protein (188 g/day) on the follicular fluid and cumulus-oocyte complexes of mid-lactating Girolando cows. Twenty-two Girolando cows with average of 21.55 ±3.19 L daily milk yield, 105.30 ±22.62 days in lactation and 3.22 ±0.03 body condition score were selected. To reduce CP in diets and maintain constant metabolizable protein, urea and soybean meal were gradually replaced by lignosulfonate-treated soybean meal (SoyPass^®^, Cargill), resulting in an increase in rumen-undegradable protein and a reduction in rumen degradable protein. A linear and quadratic reduction was observed in the plasma and follicular fluid urea nitrogen concentration following CP reduction, with the most intense reduction occurring in the 127 g Kg^-1^ DM group (p<0.001). As CP reduced, there was a tendency for a linear increase in the follicular growth rate (P=0.0696), on the number and proportion of viable oocytes (P<0.09), and also a linear increase for the number (P=0.0397) and proportion (P<0.09) of grade I viable oocytes. Plus, there was a linear effect for the number of *cumulus oophorus* cells. Cows fed with the lowest amount of CP had cumulus-oocyte complexes with higher numbers of *cumulus oophorus* cells (P=0.0238). Also, the reduction of diet crude protein was followed by a decrease in the probability of oocytes’ DNA degradation. In conclusion, the reduction of CP in the diet of mid-lactating Girolando cows, reduces urea nitrogen concentration in both blood plasma and follicular fluid, and, as a consequence, increases the viability of oocytes and the number of *cumulus oophorus* cells while reducing oocytes’ DNA degradation of follicular included cumulus-oocyte complex. The reduction on dietary CP may improve in vivo oocytes’ embryo development impacting fertility of lactating dairy cows.

## Introduction

Nutrition management is a significant factor in maximizing animal production, and an increase in crude protein (CP) or protein equivalent in the diet is directly related to an increase in milk production (Sinclair et al., 2014). When the metabolizable protein in the feed exceeds the amount recommended by the Nutritional Requirements of Dairy Cattle (NRC, 2001), an excess of nitrogen is released into the environment by urine and feces (Apelo et al., 2014). The CP content of a diet is composed for two fractions, the rumen degradable protein (RDP), that can be metabolized by ruminal microorganisms, and a rumen undegradable protein (RUP), that bypass to abomasum (NRC, 2001). The proportion of protein that delivers rumen and is made available for cow’s intestine absorption is called metabolizable protein (Bach et al., 2005). Since protein is one of the most expensive nutrients in lactating dairy cow nutrition and excreted nitrogen is an important factor in environmental pollution (Dijkstra et al., 2011), there must be an emphasis on achieving higher diet efficiency of CP.

The degradable protein fraction of a diet provides ammonia for rumen microorganisms, which use it to synthesize the microbial protein that, which is made available for cow digestion. However, diets with a high degradable protein fraction in the rumen and/or an imbalance between energy and protein ratios result in high levels of ruminal ammonia, which escapes into the blood circulation (Sinclair et al., 2000; Hristov et al., 2004). Free ammonia is toxic to the organism, thus, the liver undergoes an energy-expending process to convert it into urea, which is a less toxic and water-soluble form of nitrogen that is released into the blood. Due to its high water-solubility, urea can infiltrate the organism’s tissues until excreted in the urine by the kidneys (Bach et al., 2005). Research on Holstein cows have shown that high urea nitrogen (urea-N) in the plasma is linked to a reduction of *in vivo* and *in vitro* oocyte development competency (Santos et al., 2009; Aboozar et al., 2012; Gath et al., 2012), together with a reduction in conception rate (Lean et al., 2012).

Dairy herds in tropical and subtropical regions depend on adapted breeds to support production under an adverse environment (Porto-Neto et al., 2014). In Brazil, milk production is predominantly supported by crossbred zebu and taurine breeds, especially the Girolando - composed of Holstein (*Bos taurus*) and Gyr (*Bos indicus*) breeds - which combines Gyr’s thermal and ectoparasite tolerance benefits with the Holstein breed’s high milk production potential (Franzoni et al., 2018). Research on CP reduction in diets has focused specifically on the milk production of indoor-housed high milk production Holstein cows (Reed et al., 2017), while research on crossbred cows raised in tropical environments is still limited.

This study aimed to investigate the reduction of crude protein contents on mid-lactating Girolando cows’ diets by reducing the rumen-degradable protein in relation to rumen-undegradable protein, meeting the metabolizable protein requirements for milk production on the blood plasma, follicular fluid, oocyte-cumulus complex quality, and cell apoptosis.

## Methods

The experiment was approved by Embrapa Dairy Cattle (protocol number 14/2014) and was conducted on the corporation’s experimental farm, located in Coronel Pacheco, Minas Gerais, Brazil. The experiment’s nutritional design is further detailed in Guimarães et al. (2018). Twenty-two mid-lactating Girolando cows (ten 3/4 Holstein and twelve 7/8 Holstein, Table 1), 15 primiparous and seven multiparous, with 21.55 ±3.19 L/day daily milk production, 105.30 ± 22.62 days in lactation, 3.22 ±0.03 body condition score, and 475.8 ±7.75 Kg body weight, were used for this trial.

**Table 1:** Characteristic of treatment groups.

Treatments (g Kg^-1^ DM)	Genetic Group	Number of Cows	DIM		Milk Yield (L/day)	
156	3/4 H	3	122.33	±21.54	21.90	±0.86
	7/8 H	3	84.66	±13.20	19.31	±3.58
139	3/4 H	3	115.33	±17.15	23.90	±0.90
	7/8 H	2	105.00	±35.00	18,76	±3.48
132	3/4 H	2	113.00	±20.00	20.40	±1.19
	7/8 H	3	94.33	±4.92	20.98	±0.91
127	3/4 H	2	111.50	±18.50	22.84	±5.12
	7/8 H	4	102.00	±18.71	23.33	±2.82
Total		22	105.30	±22.62	21.55	±3.19

H: proportion of Holstein on genetic composition; DIM: days in milk. Data are average ±SD.

The animals were housed in a free-stall barn for 97 days and fed twice daily in equal proportions after milking (at 06:00 h and 15:00 h). The diets were formulated using the Cornell Net Carbohydrate and Protein System (CNCPS, version 6.1) and offered individually *ad libitum* in a Calan Broadbent Feeding System (American Calan^®^, New Hampshire, USA) with a daily adjustment to maintain orts at 10%. The first 30 days comprised of the adaptation period to the barn and the feeding system. For the final 15 days of the adaptation period, all animals received diets with 154 g CP per Kg^-1^ of DM, following the recommended nutritional requirements for this category of cows (NRC, 2001). After the adaptation period, the animals were divided into four experimental groups, with each receiving the experimental diets for the next 67 days.

The experimental diets were formulated to reduce the CP for each of the four experimental groups to 156, 139, 132, and 127 g Kg^-1^ DM, respectively. The group 156 g Kg^-1^ DM had the recommended nutritional requirements of CP (NRC, 2001). All diets maintained a constant metabolizable protein content (Pmet: 188 g/d) through a reduction in rumen-degradable protein (RDP: 97, 77, 67, and 56 g Kg^-1^ DM) in relation to rumen-undegradable protein (RUP: 57, 59, 63, and 68 g Kg^-1^ DM). The diets were formulated to be isoenergetic and composed of corn silage, ground corn, soybean meal, urea, and mineral supplement (Table 2). A soybean meal treated with lignosulfonates that prevent microbial fermentation (SoyPass^®^, Cargill^®^, Uberlândia, Brazil) was used to substitute the urea and soybean meal in the diet formulation to increase RUP (Table 2). The effect of the diets’ CP reduction on DMI, digestibility, milk yield, and nitrogen efficiency parameters of this trial are broadly outlined and discussed in Guimarães et al. (2018).

**Table 2:** Ingredients and chemical composition of experimental diets.

**Item**	**Crude protein concentration (g Kg^-1^ DM)**			
	**156**	**139**	**132**	**127**
**Ingredients (g Kg^-1^)**				
Corn silage	536	540	533	530
Corn meal	296	316	324	323
Soybean meal	133	83	70	53
Urea	87	57	29	0
Soypass^®^(1)	0	29	44	68
Sodium bicarbonate	7	7	7	7
Mineral-vitamin supplement(2)	19	19	19	19
**Chemical composition**				
Dry matter (DM, g Kg^-1^ organic matter)	582	580	583	585
Crude protein (g Kg^-1^ DM)	156	139	132	127
Gross energy (Kcal kg^-1^)	3.94	3.94	3.95	3.96
Non-fiber carbohydrate (g Kg^-1^ DM)	413	427	435	438
Neutral detergent fiber (g Kg^-1^ DM)	353	356	355	358
Acid detergent fiber (g Kg^-1^ DM)	161	161	161	162
Ether extract (g Kg^-1^ DM)	33	33	33	33
Rumen-degradable protein (g Kg^-1^ DM)(3)	97	77	67	56
Rumen-undegradable protein (g Kg^-1^ DM)^(3)^	57	59	63	68
Metabolizable protein (g/day)^(3)^	1.88	1.88	1.88	1.88
Net energy for lactation (Mcal Kg^-1^ DM)^(3)^	1.61	1.62	1.62	1.61

^(1)^Lignosulfonate-treated soybean meal (Cargill, Uberlândia, MG, Brazil). ^(2)^Composition (kilogram of dry matter): 190 g Ca, 60 g P, 20 g S, 20 g Mg, 35 g K, 70 g Na, 15 mg Co, 700 mg Cu, 10 mg Cr, 700 mg Fe, 40 mg I, 1,600 mg Mn, 19 mg Se, 2,500 mg Zn, 200,000 IU vitamin A, 50,000 IU vitamin D3,1,500 IU vitamin E, and 600 mg F. ^(3)^Estimated using the Cornell Net Carbohydrate and Protein System, version 6.1.

To measure urea-N, glucose, and non-esterified fatty acid concentrations (NEFA), blood and follicular fluid samples were harvested 22, 38, 52, and 68 days after the beginning of the experimental diets. Blood was harvested by coccygeal venipuncture via a vacuum blood collection system (Labor Import^®^, Osasco, SP, Brazil) in tubes containing sodium fluoride and EDTA (BD Vacutainer^®^, São Paulo, SP, Brazil). Follicular fluid was harvested from the largest follicle present in each animal’s ovaries (one from each animal), 120 h after wave synchronization through follicles ≥6mm ablation by ovum pick-up (OPU) (Santos et al., 2019). The blood and follicular fluid samples were cooled at 4 °C for 1 h, centrifuged (*800 x g*, 15 minutes), and the supernatant was frozen at -20 °C prior to analysis.

To ensure that the follicle punctured for fluid collection was growing and to evaluate the effect of diets on follicular growth, the two largest ovarian follicles on each ovary were measured by transrectal ultrasonography at 72 h, 96 h, and 120 h (immediately prior to fluid collection) after each follicular wave synchronization (Santos et al., 2019). The growth rate of the punctured follicle was calculated by the difference of the largest follicle diameter at 120 h from its previous diameter at 72 h and divided by three, the number of growing days (Santos et al., 2019).

Urea-N and glucose concentrations in the plasma and follicular fluid were measured, respectively, with urea UV Liquiform^®^ and Glucose Pap Liquiform^®^ biochemical kits (Labtest Diagnostica, Lagoa Santa, MG, Brazil) by ultraviolet photometry in two-point kinetics automatic equipment (Labmax Premium 240^®^, Labtest Diagnostica). The NEFA concentrations were measured by colorimetric endpoint reaction in an Eon^®^ spectrophotometer (BioTek Instruments Inc., Winooski, Vermont, USA) with a Randox^®^ NEFA assay kit (Randox Laboratories Limited, County Antrim, UK).

Cumulus-oocyte complexes (COCs) were collected by OPU 29, 33, 59, and 63 days after the experimental diets began. The COC collections were performed 72 h after wave synchronization through follicles ≥6mm ablation by OPU or after oocyte collection. Using a stereomicroscope, the COCs were scored subjectively according to cytoplasm homogeneity and *cumulus oophorus* cell layers (Viana et al., 2004). The COCs classified as Grade I, Grade II, and Grade III were considered viable, with the denuded and degenerate COCs considered non-viable.

The viable COCs collected at days 33 and 63 were fixed in 4% formaldehyde solution, kept in PVA alcohol, grouped per treatment, and stored at 4 °C. For DNA general evaluation the structures were stained with 4’6-diamidino-2-phenylindole (DAPI). DNA fragmentation was evaluated by terminal deoxynucleotidyl transferase-mediated dUTP nick end-labeling (TUNEL) stained cells, with a DeadEnd™ Fluorometric TUNEL System kit (Promega Corporation, Madison, Wisconsin, USA).

**Figure 1:**
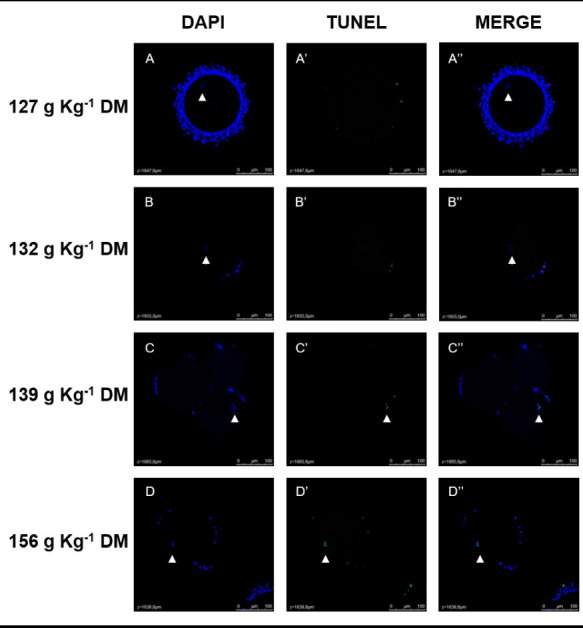
Representative images of oocytes nuclei (white arrow head) stained with DAPI (blue) and TUNEL (green) from the treatments 127g Kg^-1^ DM (A), 132 g Kg^-1^ DM (B), 139 g Kg^-1^ DM (C) and 156 g Kg^-1^ DM (D). Figures are a z-stack image showing the region of oocyte nuclei of COC for each group. Confocal fluorescence microscopy, 400 x magnification.

**Figure 2:**
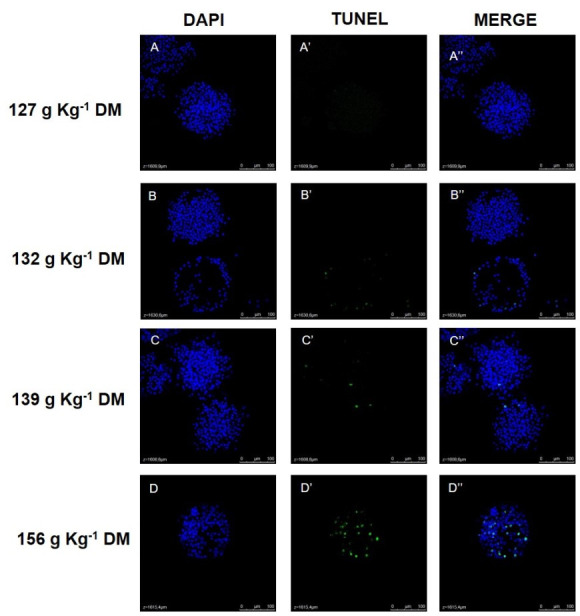
Representative images of cumulus-oocyte complexes (COC) stained with DAPI (blue) and TUNEL (green) from the treatments 127g Kg^-1^ DM (A), 132 g Kg^-1^ DM (B), 139 g Kg^-1^ DM (C) and 156 g Kg^-1^ DM (D). Figures are a z-stack image showing *cumulus oophorus* cell nuclei on the top of COC for each group. Confocal fluorescence microscopy, 400 x magnification.

The images were obtained using a Leica TCS SP5II confocal fluorescence microscope (Leica Microsystems^®^, Wetzlar, Germany), with blue filter (405 nm) to visualize the cellular nuclei and green filter (488 nm) to identify TUNEL-positive cells (Figures 1-2). Images were taken at 400x magnification, in 4 µm serial z-stacks. The images were evaluated using Leica LAS AF Lite software (Leica Microsystems^®^, Wetzlar, Germany) to identify oocytes nuclei labeled for DAPI and TUNEL and to manually count the number of *Cumulus oophorus* cells labeled for DAPI and TUNEL (Ascari et al., 2017). To avoid counting repeatedly the same *Cumulus oophorus* cell, these cells were counted manually on every four z-stack of COC (16 µm of distance). The *cumulus oophorus* TUNEL-positive index was calculated according to the ratio between the total number of TUNEL-positive cells and the total number of *cumulus oophorus* cells.

Data (except for oocyte apoptotic frequency) were analyzed as a completely randomized design using the GLIMMIX procedure of SAS (version 9.4, SAS Institute Inc., Cary, NC). Due to the quantitative nature of crude protein levels effect, orthogonal decomposition of the treatments sum of squares was used to test linear and quadratic effects. Coefficients used to build the contrasts were obtained using the IML procedure of SAS. The Kenward-Roger option was used for computing the denominator degrees of freedom for testing hypotheses. The effect of the breed was added in the model (i.e., 3/4 and 7/8 Holstein) to control this source of variation. For the variables collected on different days, the effect of day and its interaction with the crude protein levels were added to the statistical model to be evaluated as repeated measures. The best covariance structure for the models with the day of sample collection was chosen by the lowest corrected Akaike information criterion (AICc). The measurements collected at the beginning of the trial were tested as a covariable in the model. P-values ≤ 0.05 were regarded as significant. For variables non-normally distributed, the most properly probability distribution of the response variables was chosen based on the nature of the variables (e.g., count and proportions) and by using statistics of the goodness of fit as the AICc and dispersion parameter (Pearson Chi-square/degrees of freedom) (Gbur et al., 2012; Littell et al., 2006). For the oocyte apoptotic frequency, due to the binary character of this variable, a binary logistic regression was used to model the probability of positive observations as the CP concentration in the diets varied, using the GENMOD procedure of SAS (version 9.4, SAS Institute Inc., Cary, NC). The probability calculations were made according to Kaps and Lamberson (2004).

All procedures of the present study were approved by the Ethics Committee of Embrapa Dairy Cattle, Brazil, by protocol n^o^ 14/2014.

## Results

The concentrations of urea-N in the blood plasma and follicular fluid showed linear and quadratic reduction following CP concentration in the diets (P<0.0001, Table 3). The quadratic effect resulted from the intense reduction of urea-N between the groups 132 and 127 g Kg^-1^ DM (Table 3). The diets had no linear or quadratic effect on glucose and NEFA concentration in neither the blood plasma or follicular fluid (P>0.05, Table 3).

**Table 3:** Urea-N, Glucose and NEFA concentrations (LSM ±SEM) in plasma and follicular fluid from mid-lactating Girolando dairy cows fed diets with different crude protein (CP) concentrations.

**Variable**	**Treatments (g Kg^-1^ DM)**								**P-value**	
	**156 (*24*)**		**139 (*20)***		**132 (*20)***		**127 (*24)***		**Linear**	**Quad**
Urea-N (mg/dL)										
Plasma	34.54	±1.40	26.81	±1.50	24.05	±1.48	18.44	±1.38	*<0.0001*	0.0215
Follicular Fluid	28.83	±1.47	19.66	±1.65	18.23	±1.60	14.26	±1.42	*<0.0001*	0.0085
Glucose (mg/dL)										
Plasma	63.04	±1.07	64.63	±1.20	65.30	±1.18	65.29	±1.08	0.2298	0.3121
Follicular Fluid	69.60	±3.44	70.55	±4.01	71.05	±3.80	73.97	±3.42	0.3663	0.9526
NEFA (mmol/L)										
Plasma	0.061	±0.002	0.069	±0.002	0.065	±0.002	0.061	±0.002	0.4277	0.1357
Follicular Fluid	0.069	±0.006	0.065	±0.006	0.081	±0.008	0.068	±0.006	0.9897	0.2706

For glucose, the interaction between time and diets on blood plasma was affected (P=0.001), as well as tendency on follicular fluid (P=0.0741, Table 4).

**Table 4:** Glucose concentrations (LSM ±SEM) in plasma and follicular fluid based on interaction of treatments and time from mid-lactating Girolando dairy cows fed diets with different crude protein (CP) concentrations.

**Treatments (g Kg^-1^ DM)**	**Days receiving the diets**							
	**22**		**38**		**52**		**68**	
**Plasma**								
156	60.96	±1.30 (6)	62.79	±1.30 (6)	63.79	±1.30 (6)	64.63	±1.30 (6)
139	63.66	±1.43 (5)	62.26	±1.43 (5)	65.26	±1.43 (5)	67.32	±1.53 (5)
132	66.06	±1.42 (5)	66.46	±1.42 (5)	65.06	±1.42 (5)	63.66	±1.42 (5)
127	64.96	±1.31 (6)	62.62	±1.31 (6)	65.46	±1.31 (6)	68.12	±1.31 (6)
P-value								
Linear	0.0674		0.9372		0.4918		0.1494	
Quad	0.0442		0.0631		0.6498		0.3178	
**Follicular Fluid**								
156	73.12	±9.30 (6)	68.62	±4.64 (6)	67.60	±1.01 (6)	69.00	±4.05 (6)
139	65.66	±11.73 (5)	73.82	±4.79 (5)	69.08	±1.23 (5)	73.60	±4.11 (5)
132	72.64	±10.39 (5)	70.29	±5.73 (6)	69.66	±1.11 (5)	71.60	±4.11 (5)
127	70.36	±9.31 (6)	77.57	±5.01 (5)	65.97	±1.01 (6)	81.97	±3.43 (6)
P-value								
Linear	1.000		0.2674		0.1249		0.0263	
Quad	0.9417		0.8760		0.0462		0.6963	

There were no dietary effects resulting from the CP on ovaries’ follicular diameter, but there was a tendency for linear effect on follicular growth rate (P=0.0696, Table 5). As the CP on diets reduced, the follicular growth rate increased (Table 5). For recovered oocytes, there was a tendency for a linear increase in the number and proportion of viable oocytes (P<0.09) and also in the number (P<0.05) and proportion (P<0.09) of recovered Grade I viable oocytes (Table 6).

**Table 5:** Follicles diameter and follicles growth rate (LSM ±SEM) from mid-lactating Girolando dairy cows fed diets with different crude protein (CP) concentrations.

**Variable**	**Treatments (g Kg^-1^ DM)**								**P-value**	
	**156 (*24*)**		**139 (*20*)**		**132 (*20*)**		**127 (*24*)**		**Linear**	**Quad**
Follicle diameter, mm										
72h	7.25	±0.62	7.09	±0.67	7.07	±0.64	7.00	±0.67	0.8161	0.9078
96h	7.79	±0.69	8.61	±0.78	6.95	±0.75	8.47	±0.75	0.7041	0.3494
120h	8.60	±0.93	9.10	±1.06	9.40	±1.02	9.65	±1.02	0.4845	0.7490
Follicle growth rate	0.17	±0.30	0.66	±0.33	0.78	±0.32	1.09	±0.33	0.0696	0.4762

**Table 6:** Cumullus-oocyte Complex number and viability (LSM ±SEM) from mid-lactating Girolando dairy cows fed diets with different crude protein (CP) concentrations.

**Cumulus-oocyte Complexes**	**Treatments (g Kg^-1^ DM)**								**P-value**	
	**156**		**139**		**132**		**127**		**Linear**	**Quad**
Total recovered oocytes	13		85		62		115			
Recovered oocytes/cow	3.85	±1.32	4.36	±1.32	3.73	±1.32	5.83	±1.21	0.2810	0.6241
Number of viable oocytes	1.58	±0.93	2.74	±0.93	2.29	±0.92	4.03	±0.85	0.0879	0.9225
Proportion of viable oocytes	39.18	±9.7	60.35	±9.7	52.29	±9.7	68.80	±8.9	0.0794	0.7256
Number of grade I oocytes	0.23	±0.32	0.51	±0.32	0.43	±0.32	1.18	±0.29	0.0397	0.7008
Proportion of grade I oocytes	10	±6.1	11.25	±6.1	9.3	±6.80	24.58	±5.6	0.0710	0.4411
Number of grade II oocytes	0.83	±0.5	1.38	±0.5	1.28	±0.5	1.72	±0.5	0.2571	0.7922
Proportion of grade II oocytes	50.67	±12	48.25	±12	61.25	±13.5	39.12	±11	0.4801	0.3961
Number of grade III oocytes	0.52	±0.30	0.85	±0.30	0.58	±0.30	1.12	±0.28	0.2094	0.7464
Proportion of grade III oocytes	39.33	±13.6	40.5	±13.6	29.45	±15	36.3	±12.4	0.8192	0.6815

Results are LSM ±SEM.

Representative images of a z-stack of oocyte nuclei and *cumulus oophorus* cells labeled by DAPI and TUNEL for each group can be seen on Figures 1-2, respectively. A linear effect was found in *cumulus oophorus* cell numbers (Table 7). As CP content in the diets decreased, the number of cell counts increased (P<0.05). For TUNEL-positive *cumulus oophorus* cells, a linear tendency was recorded (P=0.05) and a quadratic effect (P=0.0449) as CP content was reduced. While the group 139 g Kg^-1^ DM group had the biggest number of TUNEL-positive cells, for the 127 g Kg^-1^ DM group these cell numbers were the smallest (Table 7). However, there was no significant effect found on the *cumulus oophorus* cell TUNEL-positive index (P>0.05, Table 7). Representative images of oocyte and *cumulus oophorus* cells nuclei labeled by DAPI and TUNEL for each treatment are presented in Figure 1.

**Table 7:** *Cumulus oophorus* cells number, apoptotic *cumulus oophorus* cells and *cumulus oophorus* cells apoptotic index (LSM ±SEM) from mid-lactating Girolando dairy cows fed diets with different crude protein (CP) concentrations.

***Cumulus oophorus* cells**	**Treatments (g Kg^-1^ DM)**								**P-value**	
	**156 (*3*)**		**139 (*19*)**		**132 (*19*)**		**127 (*34*)**		**Linear**	**Quad**
Total number	175.67	±102.06	308.30	±67.1	162.49	±38.58	589.10	±108.32	0.0238	0.3500
TUNEL-positive										
Number	3.67	±4.05	28.46	±10.54	10.71	±4.35	1.39	±0.6	0.0500	0.0449
Index	0.08	±0.054	0.10	±0.026	0.14	±0.036	0.06	±0.03	0.5616	0.2333

**Figure 3:**
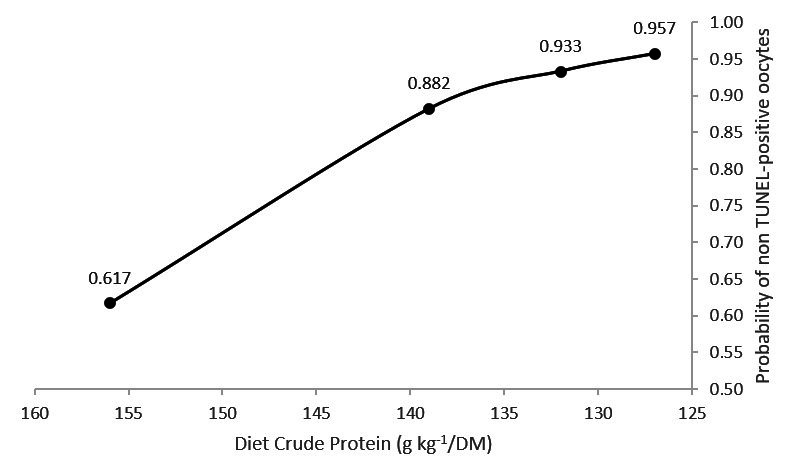
Probability of non TUNEL-positive oocytes nuclei DNA in response to the variation in the crude protein contents of mid-lactating Girolando dairy cows.

As the dietary CP content decreased the probability of observing a TUNEL-positive oocyte also decreased significantly (Figure 3). The binary logistic regression resulted in the following coefficients: β_0_ = 14.5466 (P=0.027) and β_1_ = -0.0902 (P=0.060).

## Discussion

Although studies have been associating high CP diets with reduced fertility in dairy cows, the reasons have not yet been well understood (Butler, 1998; Sawa et al., 2011; Sinclair et al., 2014). Nevertheless, reducing the levels of dietary protein can compromise milk production (Sinclair et al., 2014).

Despite the CP reduction in their diets, all the mid-lactating Girolando cows in this study were able to intestinally absorb the same amount of protein once the diets were balanced to supply the same metabolizable protein between the groups. Additionally, as shown in our previous paper (Guimarães et al., 2018), the reduction of CP resulted in improved nitrogen efficiency, thus, reducing urinary nitrogen excretion and milk urea nitrogen, without altering the DMI and milk production.

The follicular fluid makes up the biochemical environment where the oocytes develop until ovulation and is composed of the filtrated blood plasma associated with the molecules produced by granulosa cells and oocytes (Leroy et al., 2004; Rodgers and Irving-Rodgers, 2010). Many factors affect its composition, including the cow’s physiological and nutritional status, which may also affect the oocyte development competency (Sales et al., 2015). The similar variation pattern observed for urea-N, glucose and NEFA concentrations between the blood plasma and follicular fluid were expected, due to blood metabolite concentrations having a high correlation with metabolites found in the follicular fluid (Leroy et al., 2004; Ribeiro et al., 2020).

The reduction of CP in the diets of mid-lactating Girolando cows resulted in both the linear and quadratic reduction of nitrogen ureic on blood plasma and follicular fluid resulting from the intense reduction observed on the group 127 g Kg^-1^ DM group. Reduction in plasma urea-N was expected due to a reduction of protein availability for ruminal degradation, although Aboozar et al. (2012) did not find a difference between the early post-partum Holstein cows fed with lower CP diets based on increasing the RUP percentage. However, similar to the present study, a reduction in plasma and follicular fluid urea-N was observed for Holstein heifers following a dietary reduction of CP (Moallem et al., 2011). Our data support this previous find showing that a reduction of CP availability to ruminal degradation can reduce urea-N concentration in the blood plasma and, consequently, in the follicular fluid of cows with reduced metabolic demand, such as crossbred mid-lactating cows.

Similar to other studies featuring Holstein heifers (Moallem et al., 2011) and early lactating Holstein cows (Bahrami-Yekdangi et al., 2014), the concentrations of Glucose and NEFA on plasma and follicular fluid showed no significant variation between groups. Besides the variation of glucose on plasma and follicular fluid among the interactions between treatment and day of sample collection, glucose concentrations were between physiological limit and NEFA concentration under the minimum limit for lactating dairy cows (Ribeiro et al., 2020). Clearly the metabolizable protein applied to the four diets supported the energy demands that the mid-lactating Girolando cows required for maintenance and milk production, regardless of CP reduction.

DNA damage identified by TUNEL staining is associated with the late stages of oocytes apoptosis and can impair its embryo development capability (Li et al., 2009). The groups with the highest urea-N in the follicular fluid had a greater probability of having follicule included oocytes with DNA degradation. McCormick et al. (1999) observed a reduction of 22 percentual points in pregnancy rate of Holstein cows when the blood urea-N concentration was above 25 mg/dL. Herein, the two groups with the highest dietary CP had an average plasma urea-N above the upper limit of 25 mg/dL and the highest probability for DNA degradation of oocyte nuclei. Gath et al. (2012) suggested that the effect of urea-N on fertility takes place at either the early oocyte development stages or during fertilization, as there was no effect of high urea diets of recipients cows on the pregnancy rates of beef cow after in vitro embryo transfer. The present study’s data suggest that the DNA degradation of follicular-included oocytes may act as a mechanism via which urea-N from ruminal protein degradation can disrupt cows’ fertility.

The subjective score classification of cumulus-oocyte complexes based on cytoplasm characteristics and the subjective number of *cumulus oophorus* cell layers is routinely used as an oocyte development competency indicator (Yuan et al., 2005). The CP reduction increased the number and proportion of Grade I viable oocytes recovered by OPU. Plus, the confocal analysis of COC showed that the group with the lowest dietary CP on diet had the highest number of *cumulus oophorus* cells and a reduced number of TUNEL-positive *cumulus oophorus* cells.

The literature supports the hypothesis that high diet crude protein can reduce cow fertility due to the increase of urea metabolites in the blood (Santos et al., 2009; Aboozar et al., 2012; Gath et al., 2012). In the present study, the reduction of CP concentration on mid-lactating Girolando cows’ diets was followed by a drop in the urea-N concentrations in the blood and in the follicular fluid, without affecting the energy metabolism parameters measured. Besides the reduction in the probability of oocyte nuclei DNA degradation, the reduction on blood and follicular fluid urea-N was also followed by an increase in follicular growth rate, the proportion of viable Grade I oocytes, an increase in the number of *Cumulus oophorus* cells, and a reduction of *Cumulus oophorus* cells apoptosis. Our results clearly show that free urea originating from rumen protein degradation can impair follicular cells mitosis and be toxic to the follicle inducing DNA degradation during follicular development, indicating that this may be a mechanism via which higher urea nitrogen can impair dairy cows’ fertility.

## Conclusion

Reduction of diet crude protein on mid-lactating Girolando cows’ diet while meeting adequate metabolizable protein requirements changes the intra-follicular environment by reducing follicular fluid urea-N without impairing energy metabolism. Furthermore, the reduction of urea-N is followed by an increase in oocyte quality and a reduction of DNA degradation of cumulus-oocyte complexes.
